# Natural history museum collection and citizen science data show advancing phenology of Danish hoverflies (Insecta: Diptera, Syrphidae) with increasing annual temperature

**DOI:** 10.1371/journal.pone.0232980

**Published:** 2020-05-13

**Authors:** Kent Olsen, Thomas Eske Holm, Thomas Pape, Thomas J. Simonsen

**Affiliations:** 1 Natural History Museum Aarhus, Aarhus C, Denmark; 2 Department of Bioscience, Aarhus University, Rønde, Denmark; 3 Natural History Museum of Denmark, Copenhagen, Denmark; University of Hyogo, JAPAN

## Abstract

We explore the phenological response by Danish hoverflies (Syrphidae) to continually rising annual temperatures by analysing >50.000 natural history collection and citizen science records for 37 species collected between 1900 and 2018, a period during which the annual average temperature in Denmark rose significantly (p << 0.01). We perform a simple linear regression analysis of the 10^th^ percentile observation date for each species against year of observation. Fourteen of the species showed a statistically significant (p < 0.05) negative correlation between 10^th^ percentile date and year of observation, indicating earlier emergence as a likely response to climatic warming. Eighteen species showed a non-significant (p ≥ 0.05) negative correlation between 10^th^ percentile date and year of observation, while four species showed a non-significant (p ≥ 0.05) positive correlation, and one showed neither a positive nor a negative correlation. We explore the possible impact of the length of the data series on the regression analysis by dividing the species into four groups depending on how far back in time we have data: ultra-short series (with data from 2003–2018); short series (data from 1998–2018); medium series (data from 1980–2018); long series (data from 2018 to before 1980). The length of the series seems to have an effect on the results as 60% of the long series species (nine out of 15) showed a statistically significant negative correlation, while for the shorter series species less than 35% showed a statistically significant negative correlation. When we reduced the long series in length to short series, the proportion of statistically significant negative correlations fell to 33%, confirming this assumption. We conclude that northern temperate hoverflies generally react to the ongoing climatic warming by emerging earlier.

## Introduction

It is widely acknowledged that insects respond rapidly to climatic and environmental changes [e.g., 1–4]. Such responses include phenological changes [e.g., 2, 3, 5–8], range and community shifts [9–11], phenotypical changes [12], and even maladaptations like increased cannibalism [13]. While some changes such as range expansion or contraction often are relatively easy to detect, monitor and document, others such as changes in abundance, behaviour and phenology often are more subtle and harder to document—not least because historical baseline data often are missing, forcing researchers to rely on anecdotal evidence [e.g., 1, 14, 15].

For some taxonomic groups, popular through the ages with insect collectors and more recently insect observers and photographers, natural history museum collections can provide such baseline data, at least for certain types of changes [16, 17]. Such groups include dragonflies (Odonata), bees (Hymenoptera: Anthophila), butterflies (Lepidoptera: Papilionoidea) and hoverflies (Diptera: Syrphidae), which have been the subjects of considerable interest from collectors for more than a century and today are popular amongst amateur naturalists, who report their observations to various national and international social networks and online databases. While phenological shifts in dragonflies, bees and butterflies have been demonstrated based on both museum collections and citizen science data [2, 3, 5, 6, 8, 18] only two studies have thus far focused on hoverflies [7, 19].

Hoverflies are especially interesting from this perspective; not only is the imago of the different species often conspicuous and relatively easy to observe and identify, but their various life cycle adaptations represent a range of functional traits. The imagoes are important pollinators in different natural habitats [19–21], whereas they employ a wide range of adaptations in their larval lifestyles such a predators, herbivores, coprophages, detritivores, terrestrial saprotrophs, and aquatic saprotrophs, and therefore provide an excellent opportunity to explore changes across various ecological lifestyle traits [22].

In this study, we use data from natural history museum collections and a citizen science database to test for correlation between earliest recorded flight date each year and year of observation for a selected number of Danish hoverfly species representing a range of different lifestyles. If hoverfly species emerge or arrive earlier in response to an increase in temperature, we would expect a negative correlation between earliest flight date and year of observation. Furthermore, we evaluate whether length of the data series influences the usefulness of the data.

## Materials and methods

### Data

Phenological data based on collection date or observation date (hereafter combined as observation date) of the imago (Table 1) were obtained from two main data sources: 1) digitised museum collection data from the Natural History Museum Aarhus (NHMA) and the Natural History Museum of Denmark (NHMD), which represent the two largest public entomological collections in Denmark, and 2) citizen science data with rigorous quality control of observations from the ongoing Atlas of Danish Hoverflies (https://www.svirreflueatlas.dk/). The atlas project is part of Naturbasen (https://www.naturbasen.dk/), which is the largest citizen science portal in Denmark and has been in use since 2001.

**Table 1:** Species of Danish hoverflies included in the study with information on biology, number of specimens included (n), relative length of series, and statistical p-values. Other regression analyses values including 95% confidence intervals are provided in S1 Data.

Species	Larval biology	n	Migratory	Data series	Correlation DOY/year	p-value	Slope coeff.	R^2^ coeff.	Reduced series p-value
*Anasimyia lineata*	Saprophage, aquatic	474	No	Medium*	Negative	0.1109	-0.1729	0.1114	
*Arctophila superbiens*	Saprophage, aquatic	444	No	Short	Positive	0.4866	0.5459	0.0450	
*Cheilosia pagana*	Herbivore, internal	1351	No	Long	Negative	**5.9E-05**	-0.4263	0.2734	≥ 0.05
*Chrysotoxum bicinctum*	Predator, aphids	487	No	Ultra short	Negative	**0.0312**	-0.7864	0.3854	
*Episyrphus balteatus*	Predator, aphids	6093	Primary	Long	Negative	**8.92E-05**	-0.5879	0.1679	≥ 0.05
*Eristalinus sepulchralis*	Saprophage, aquatic	666	No	Long	Negative	**1.88E-06**	-0.4075	0.6037	**< 0.05**
*Eristalis arbustorum*	Saprophage, aquatic	1122	Primary	Short	Negative	0.1023	-2.7481	0.2241	
*Eristalis interrupta*	Saprophage, aquatic	549	No	Short	Negative	0.0552	-2.1869	0.2731	
*Eristalis intricaria*	Saprophage, aquatic	1181	No	Ultra short	Negative	0.3736	-1.1306	0.0665	
*Eristalis lineata*	Saprophage, aquatic	715	No	Short*	Negative	0.1091	-2.2209	0.1730	
*Eristalis pertinax*	Saprophage, aquatic	1591	No	Short	Negative	0.0845	-0.6090	0.1485	
*Eristalis tenax*	Saprophage, aquatic	3681	Primary	Long	Negative	**1.36E-07**	-0.9171	0.2918	≥ 0.05
*Eupeodes corollae*	Predator, aphids	2807	Primary	Long	Negative	0.0532	-0.2055	0.0477	≥ 0.05
*Helophilus hybridus*	Saprophage, aquatic	406	No	Ultra short	Positive	0.2147	1.4164	0.1493	
*Helophilus pendulus*	Saprophage, aquatic	5196	No	Long	Negative	**0.0029**	-0.2154	0.0996	**< 0.01**
*Helophilus trivittatus*	Saprophage, aquatic	892	No	Ultra short	Negative	0.1149	-2.0455	0.2297	
*Melangyna lasiophthalma*	Predator, aphids	430	No	Medium	Negative	**0.0163**	-0.5768	0.2451	
*Melanostoma mellinum*	Predator, aphids	737	No	Ultra short	Negative	**0.0213**	-2.2904	0.3953	
*Melanostoma scalare*	Predator, aphids	721	No	Ultra short	Positive	0.6703	0.2189	0.0171	
*Meliscaeva cinctella*	Predator, aphids	780	No	Medium*	None	0.9974	-0.0007	4E-07	
*Merodon equestris*	Herbivore, internal	795	No	Short	Negative	**0.0061**	-1.0385	0.4258	
*Myathropa florea*	Saprophage, aquatic	2366	No	Long	Negative	**0.0272**	-0.1410	0.0718	≥ 0.05
*Rhingia campestris*	Coprophage	1735	No	Long	Negative	**8.49E-06**	-0.3380	0.2793	≥ 0.05
*Scaeva pyrastri*	Predator, aphids	2038	Obligate	Long	Negative	**0.0123**	-0.1816	0.0818	**< 0.01**
*Scaeva selenitica*	Predator, aphids	487	Primary	Medium	Negative	0.3026	-1.3701	0.0530	
*Sericomyia silentis*	Saprophage, aquatic	1604	No	Medium*	Negative	0.0770	-0.2802	0.1299	
*Sphaerophoria scripta*	Predator, aphids	1199	No	Long	Negative	0.5641	-0.0746	0.0112	≥ 0.05
*Syritta pipiens*	Saprophage, terrestrial	1102	No	Short	Negative	0.3193	-0.4083	0.0762	
*Syrphus ribesii*	Predator, aphids	1175	No	Long	Negative	0.0727	-0.3173	0.1034	≥ 0.05
*Syrphus torvus*	Predator, aphids	1465	No	Long	Negative	0.4612	-0.1200	0.0091	**< 0.01**
*Syrphus vitripennis*	Predator, aphids	1034	No	Long	Negative	0.4173	-0.2700	0.0228	**< 0.01**
*Tropidia scita*	Saprophage, aquatic	435	No	Medium	Negative	**0.0275**	-0.5292	0.2204	
*Volucella bombylans*	Saprophage, Hymenoptera nests	1235	No	Long	Negative	**0.0005**	-0.2362	0.3559	≥ 0.05
*Volucella pellucens*	Saprophage, Hymenoptera nests	2866	No	Long	Negative	0.2915	-0.0689	0.0157	≥ 0.05
*Xanthogramma pedissequum*	Predator, aphids	425	No	Ultra short	Negative	0.1945	-1.5669	0.1479	
*Xylota segnis*	Saprophage, terrestrial	856	No	Ultra short	Positive	0.2095	0.7154	0.1391	
*Xylota sylvarum*	Saprophage, terrestrial	454	No	Ultra short	Negative	0.1736	-1.2382	0.1614	

We selected Danish hoverfly species to be included in the analysis based on the following criteria: 1) they represent a broad selection of the biology and phenology known from Danish hoverfly species, 2) they provide a broad taxonomic coverage of the Danish hoverfly fauna, and 3) it is possible for skilled amateurs to identify them correctly in the field. Observation dates for 37 species of hoverflies were included in the analyses (Table 1).

Observation dates for ten species originated from all three data sources, data for 22 species from NHMA and Naturbasen, while data for the remaining 15 species originated from Naturbasen only. Observation dates were converted into day of the year (DOY), i.e., January 1^st^ corresponds to DOY 1 etc. Data from the museum collections were limited to the 20^th^ and 21^st^ centuries as data from before 1900 are very limited. Life history data were adopted from [23–25]. The largest data series (*Episyrphus balteatus*) comprises 6.093 observation dates, while the shortest data series (*Helophilus hybridus*) comprises 406 observation dates. The raw data set comprises 51.595 observation dates with an average of 1.097 observations per species. The full data set is available as S1 Data.

Denmark is a small and climatically homogenous geographical area, allowing us to pool all data rather than divide data points into regional subsets, and thereby avoid undermining the statistical power of the data.

### Analysis

As we seek to explore whether there is a change in the earliest flight date for Danish hoverflies as a possible response to a general increase in temperature, we first performed a simple linear regression analysis of the annual mean temperature against the year for the period 1900–2018 ([26]; all underlying temperature data are available from the Danish Meteorological Institute: https://www.dmi.dk/publikationer/).

To minimise sampling bias and to obtain more robust correlation data, we followed [3] and used the 10^th^ percentile date for each year instead of the actual earliest observation date. Before calculating the 10^th^ percentile date, we removed duplicate DOY records and all years with fewer than three individual DOY records from the dataset for each species so each year comprise unique DOYs only. We plotted the 10^th^ percentile DOY for each species against year of observation and added a trend line. We then performed a linear regression analysis to determine if any correlation was statistically significant. Outputs from the regression analyses and the datasets used for the analyses are available in S1 Data. All analyses were performed in Microsoft Excel 2013^®^.

To explore whether the length of the data series influenced the results, we divided the species into four different groups: ultra-short series (records limited to the past 16 years: 2003–2018); short series (records limited to the past 20 years: 1998–2018); medium length series (records limited to the past 38 years: 1980–2018); long series (including pre-1980 records). Four species have a few observation dates dating further back than the group to which they were assigned (marked with an asterisk [*] in Table 1). However, these represented single data points rather than a continuum, and we therefore included them in the analyses, but not in the group assignment.

## Results

### Overall

**Fig 1:**
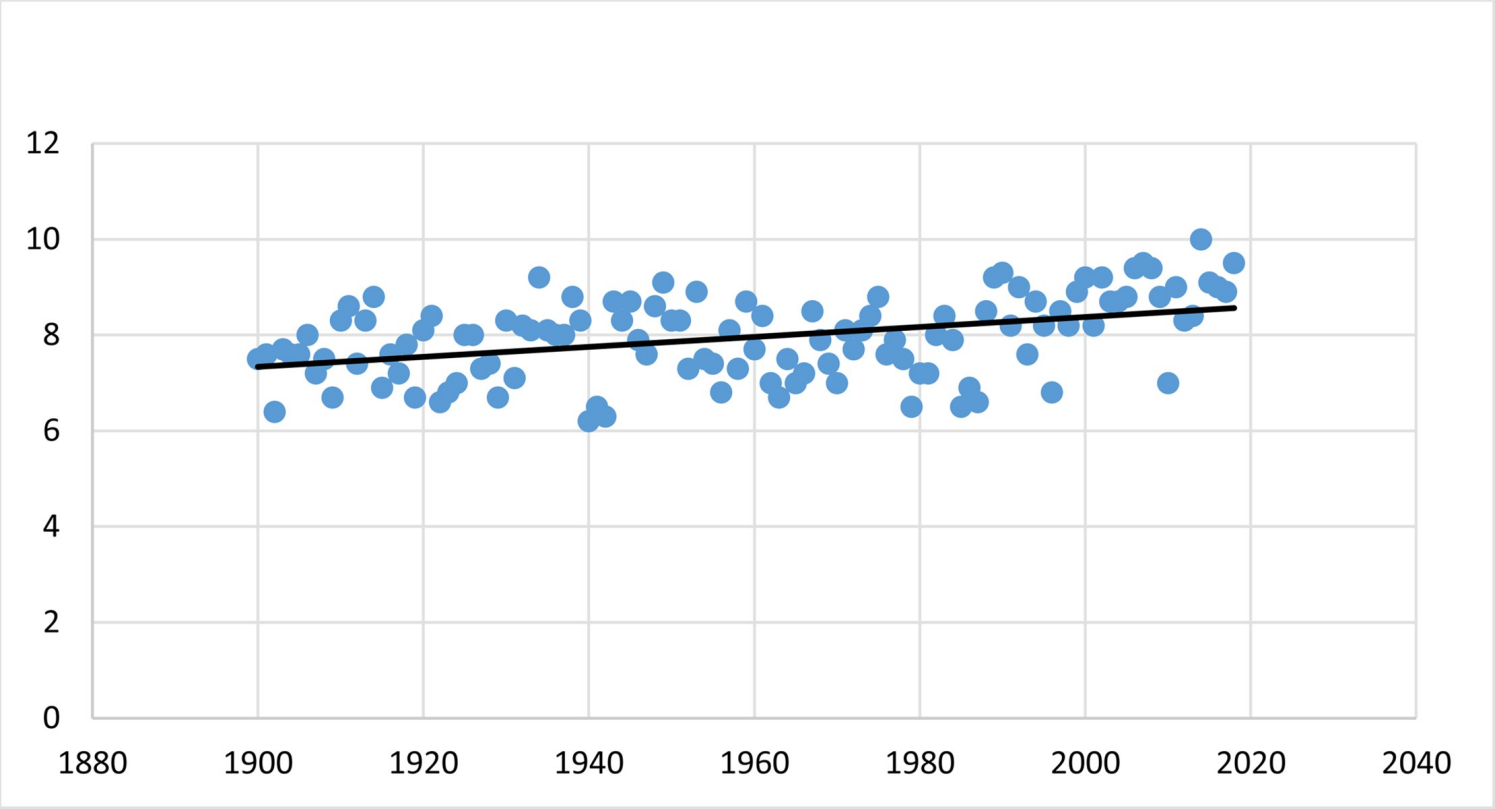
Trend line plot of the annual average temperature (y-axis) in Denmark between 1900 and 2018 (x-axis), based on [26]: p = 1.54E-06; R^2^ = 0.1798; slope coeff. = 0.0104.

The annual mean temperature in Denmark has increased significantly with almost exactly 0.01°C per year between 1900 and 2018 (R^2^ = 0.1798; p < 0.001; Fig 1, [26]).

**Fig 2:**
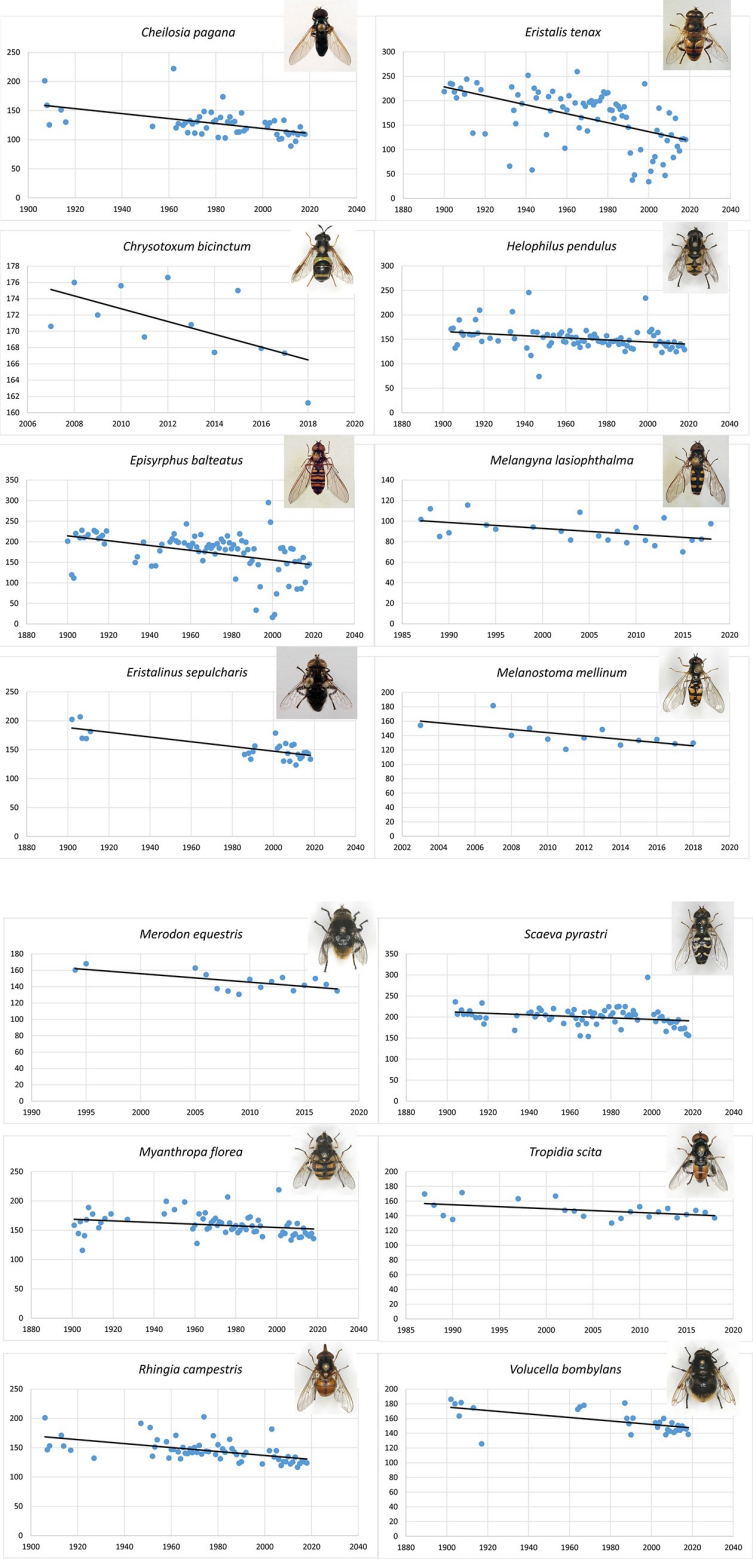
(**A**)Trend line plots of 10^th^ percentile DOY (y-axis) and year (x-axis) for eight species of Danish hoverfly species with a statistically significant correlation between the recorded earliest flight date and the year of observation. p-values, slope coefficient-values and R^2^-values from the regression analyses are given in Table 1. (**B)** Trend line plots of 10^th^ percentile DOY (y-axis) and year (x-axis) for six species of Danish hoverfly species with a statistically significant correlation between the recorded earliest flight date and the year of observation. p-values, slope coefficient-values and R^2^-values from the regression analyses are given in Table 1.

Four species displayed a positive correlation and 32 species displayed a negative correlation between the 10^th^ percentile DOY and year of observation. One species showed no correlation as demonstrated by a flat trend line (slope coefficient = 0.001). In 14 of the species with a negative correlation, the relationship was statistically significant with p < 0.05. In all other species, the correlation (negative or positive) was not statistically significant. The results are summarised in Table 1, and plots with trend lines for the 14 species with statistically significant correlations are illustrated in Fig 2, while the remaining 23 species with non-significant (or no) correlations are shown in S1 Fig.

### Biological aspects

#### –Effect of juvenile lifestyle

The two major larval lifestyles in our dataset are 1) aquatic saprophages (15 species), and 2) aphid predators (14 species). For both lifestyles, the fraction of species that displayed a significant negative correlation between the 10^th^ percentile DOY and year of observation (5/15 and 5/14, respectively) is similar to the overall observed pattern. Other lifestyles include herbivorous internal feeder (2 species displaying significant negative correlation/2 species), saprophagous in the nests of social Hymenoptera (1/2), terrestrial saprophagous (0/3), and coprophagous (1/1) (Table 1).

#### –Migratory species

Six species are migratory from Central or southern Europe, either primarily or obligate. Of these, three species (50%) displayed a significant negative correlation between the 10^th^ percentile DOY and year of observation.

### Effects of data series length

The length of the data series seems to have an effect on whether an observed correlation is statistically significant: data series for 15 species were classified as long, of these nine (60%) displayed a statistically significant correlation between the 10^th^ percentile DOY and year of observation. For species classified with medium, short, and ultra-short data series, the proportions were 2/6 (33%), 2/7 (29%), and 2/9 (22%). When long data series were reduced to short series, the proportion fell to 5/15 (33%).

### Average phenological shift

The 14 species, which displayed a statistically significant negative correlation between the 10^th^ percentile DOY and year of observation, showed on average an earliest recorded flight date estimated to be 11.1 days earlier in 2018 as compared to 2000. However, this estimated average covers a considerable variation ranging from 2.5 days (95% confidence interval: 0.3–4.8 days) in *Myathropa florea* to 41.2 days (95%: 7.4–75.1 days) in *Melanostoma mellinum*. Interestingly, the two species in which we observed the largest shift: *M*. *mellinum*, and *Merodon equestris* (18.5 days (95%: 6.2–31.1 days)), are species for which we had only short or ultra-short data series. In contrast, *Eristalis tenax* (16.5 days (95%: 10.8–22.3 days)) is one of the species for which we have the most comprehensive data series.

## Discussion

Although we only found a significant negative correlation between the 10^th^ percentile DOY and year of observation in 14 of 37 examined species of Danish hoverflies, the true number is probably considerably higher, since 32 of the 37 species displayed a negative correlation. We only had long data series for 14 of the 37 species, and in nine of these species, the negative correlation was statistically significant. It is thus likely that a larger number of the species would display a significant correlation between the 10^th^ percentile DOY and year of observation if more of our data series had been long. This assumption is supported by the fact that when we reduced the long data series to short series comprising only data from 1998–2018, the number of species with statistically significant correlations dropped from nine to three, and in one of the cases that remained significant, the p-value rose from p < 0.01 to p < 0.05. However, two of the five species that displayed a statistically significant negative correlation when the long data series was reduced, did not show a significant correlation for the original long series, but only when the series were shortened to comprise data from 1998–2018.

Our results support earlier works on hoverflies [7, 19], dragonflies [6], bees [2] and butterflies [3, 5, 8, 18] which reported that species are emerging significantly earlier in Northern Hemisphere temperate regions, than they did in the past, and that this is correlated with a rising average annual temperature. Interestingly, [7] found significantly earlier emergence for both *Er*. *tenax* and *Er*. *pertinax*, whereas we only found the pattern to be significant for the former and not for the latter. However, the p-value returned for the latter was 0.0845, which is only marginally above 0.05 normally considered the threshold for statistical significance.

Similarly to [19], we do not find any relationship between larval lifestyle and shift in phenology. It does, however, seem as if migratory species tend to be observed earlier than non-migratory species, i.e., react stronger on the documented climate change, as 60% of the primarily migratory species in our data set are observed significantly earlier, compared to 32% of the non-migratory species. One explanation for this could be that as the migratory species are already active further south in Europe, they can react more promptly and migrate north as soon as the temperature is suitable. Some migratory species such as *Episyrphus balteatus* and *Eristalis tenax* also hibernate as adults, which further shortens the time needed to react to rising temperatures.

Between 2000 and 2018, the 14 species with significant negative correlations have on average advanced their phenology by more than 11 days, while the average annual temperature in Denmark has risen by less than 0.25°C. This means that the phenological advance on average is greater than 40 days °C^-1^, a much higher ratio than the 6–10 days °C^-1^ previously reported for butterflies [3, 5, 8], and bees [2].

The fact that we find by far the greatest ratio of statistically significant results among the species where we had access to long, detailed data series illustrates the importance of well-maintained and continuously expanded natural history museum collections, as well as solid citizen science data, particularly in periods of dramatic changes in both climate and biodiversity (see also [17]).

Phenological changes in important pollinators such as hoverflies could lead to a phenological mismatch between the pollinators and the plants they pollinate [21, 27]. However, the potential effects of such a mismatch are poorly understood, although it may result in reduced reproductive success for the plant [4]. Still, phenological mismatches in mutualistic systems are expected to be short-lived, as the mutualists are under strong selection pressure to resynchronise their phenology. It is unclear how this could affect Danish and Scandinavian hoverfly-plant interactions, as knowledge of floral specialization in Scandinavian hoverfly pollinators is mostly anecdotal, underlining the need for detailed natural history studies even in some of the most well-explored regions of the world.

## Supporting information

S1 Data Raw observation data, DOY dates, and statistical analyses output for each species included in this study. (XLSX)

S1 Fig **Trend line plots of 10**^**th**^ **percentile DOY (y-axis) and year (x-axis) for the 22 species of Danish hoverflies, which did not show a statistically significant correlation between the recorded earliest flight date and the year of observation.** p-values, slope coefficient-values and R^2^-values from the regression analyses can be found in Table 1.
(PDF)

## References

[1] Thackeray SJ, Sparks TH, Frederiksen M, Burthe S, Bacon PJ, Bell JR, et al Trophic level asynchrony in rates of phenological change for marine, freshwater and terrestrial environments. Glob Chang Biol. 2010; 16: 3304–3313.

[2] Bartomeus I, Ascher JS, Wagner D, Danforth BN, Colla S, Kornbluth S, et al Climate-associated phenological advances in bee pollinators and bee-pollinated plants. Proc Natl Acad Sci U S. 2011; 108: 20645–20649. 10.1073/pnas.1115559108 PMC3251156 22143794

[3] Brooks SJ, Self A, Toloni F, Sparks T. Natural history museum collections provide information on phenological change in British butterflies since the late-nineteenth century. Int J Biometeorol. 2014; 58: 1749–1758 10.1007/s00484-013-0780-6 24429705

[4] Forrest JRK. Complex responses of insect phenology to climate change. Curr Opin Insect Sci. 2016; 17: 49–54. 10.1016/j.cois.2016.07.002 27720073

[5] Roy DB, Sparks TH. Phenology of British butterflies and climate change. Glob Chang Biol. 2000; 6: 407–416.

[6] Dingemanse NJ, Kalkman VJ. Changing temperature regimes have advanced the phenology of Odonata in the Netherlands. Ecol Entom. 2008; 33: 394–402.

[7] Graham-Taylor LG, Stubbs AE, Brooke ML. Changes in phenology of hoverflies in a central England garden. Insect Conserv Divers. 2009; 2: 29–35.

[8] Polgar CA, Primack RB, Williams EH, Stichter S, Hitchcock C. Climate effects on the flight period of Lycaenid butterflies in Massachusetts. Biol Conserv. 2013; 160: 25–31

[9] Walther GR, Post E, Convey P, Menzel A, Parmesan C, Beebee TJC. Ecological responses to recent climate change. Nature. 2002; 416: 389–395. 10.1038/416389a 11919621

[10] Thomsen PF, Jørgensen PS, Bruun HH, Pedersen J, Riis-Nielsen T, Jonko K, et al Resource specialists lead local insect community turnover associated with temperature–analysis of an 18-year full-seasonal record of moths and beetles. J Animal Ecol. 2015; 85: 251–261. 10.1111/1365-2656.12452 26521706

[11] Burley HM, Mokany K, Ferrier S, Laffan SW, Williams KJ, Harwood TD. Macroecological scale effects of biodiversity on ecosystem functions under environmental change. Ecol Evol. 2016; 6: 2579–2593. 10.1002/ece3.2036 27066246 PMC4798165

[12] Bowden JJ, Eskildsen A, Hansen RR, Olsen K, Kurle CM, Høye TT. High-Arctic butterflies become smaller with rising temperatures. Biol. Lett. 2015; 11: 20150574 10.1098/rsbl.2015.0574 26445981 PMC4650173

[13] Start D, Kirk D, Shea D, Gilbert B. Cannibalism by damselflies increases with rising temperature. Biol Lett. 2017; 13: 20170175 10.1098/rsbl.2017.0175 28515331 PMC5454245

[14] Harrington R, Woiwod IP, Sparks TH. Climate change and trophic interactions. Trends Ecol Evol. 1999; 14: 146–150. 10.1016/s0169-5347(99)01604-3 10322520

[15] Janzen D.H. & Halwachs W. Perspective: Where might be many tropical insects? Biological Conservation. 2019; 233: 102–108. 10.1016/j.biocon.2019.02.030

[16] Pyke GH, Ehrlich PR. Biological collections and ecological/ environmental research: a review, some observations and a look to the future. Biol Rev. 2010; 85: 247–26. 10.1111/j.1469-185X.2009.00098.x 19961469

[17] Johnson K, Brooks SJ, Fenberg P, Glover A, James K, Lister A, et al Climate change and biosphere response: unlocking the collections vault. BioScience. 2011; 61: 147–153.

[18] Sparks TH, Yates TJ. The effect of spring temperature on the appearance dates of British butterflies 1883–1993. Ecography. 1997; 20: 368–374

[19] Hassel C, Owen J, Gilbert F. Phenological shifts in hoverflies (Diptera: Syrphidae): linking measurement and mechanism. Ecography. 2017; 40: 853–863.

[20] Memmott J, Craze PG, Waser NM, Price MV. Global warming and the disruption of plant–pollinator interactions. Ecol Lett. 2007; 10: 710–717. 10.1111/j.1461-0248.2007.01061.x 17594426

[21] Iler AM, Inouye DW, Høye TT, Miller-Rushing AJ, Burkle LA, Johnston EB. Maintenance of temporal synchrony between syrphid flies and floral resources despite different phenological responses to climate. Glob Chang Biol. 2013; 19: 2348–2359. 10.1111/gcb.12246 23640772

[22] Rotheray GE, Gilbert F. The Natural History of Hoverflies. Forrest Text, Ceredigon, UK; 2011.

[23] Torp E. Danmarks Svirrefluer (Diptera: Syrphidae). Danmarks Dyreliv, vol. 6, Apollo Books, Stenstrup, Denmark; 1994.

[24] Bartsch H, Binkiewicz E, Rådén A, Nasibov E. Nationalnycklen til Sveriges flora och fauna. Tvåvinger: Blomflugor. Diptera: Syrphidae: Syrphinae ArtDatabanken, SLU, Uppsala, Sweden; 2009.

[25] Bartsch H, Binkiewicz E, Klintbjer A, Rådén A, Nasibov E. Nationalnycklen til Sveriges flora och fauna. Tvåvinger: Blomflugor. Diptera: Syrphidae: Eristalinae & Microdontinae ArtDatabanken, SLU, Uppsala, Sweden; 2009.

[26] Cappelen J, Kern-Hansen C, Lauersen EV, Jørgensen PV, Jørgensen BV. Denmark—DMI Historical Climate Data Collection 1768–2018. DMI rapport 19–02. 2019; Danmarks Meteorologiske Institut. Available at: https://www.dmi.dk/fileadmin/user_upload/Rapporter/TR/2019/DMIRep19-02.pdf Data available at: https://www.dmi.dk/publikationer/

[27] Renner SS, Zohner CM. Climate change and phenological mismatch in trophic interactions among plants, insects, and vertebrates. Annu Rev Ecol Evol Syst. 2018; 49: 165–182.

