# Supplementary figures and images for: Natural history museum collection and citizen science data show advancing phenology of Danish hoverflies (Insecta: Diptera, Syrphidae) with increasing annual temperature
Source: PLoS One. 2020 May 13;15(5):e0232980. doi: 10.1371/journal.pone.0232980 (PMC7219768; doi:10.1371/journal.pone.0232980)

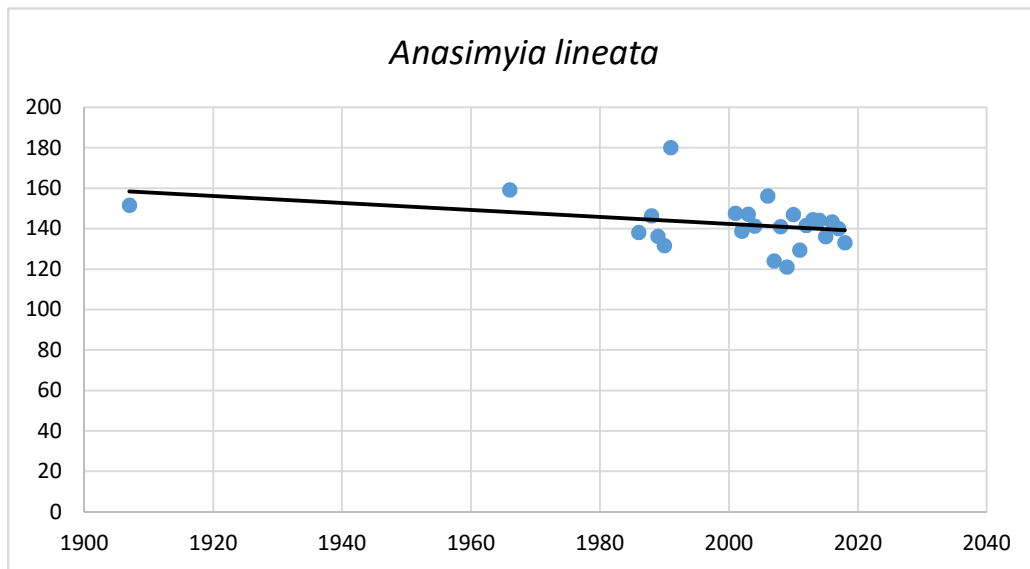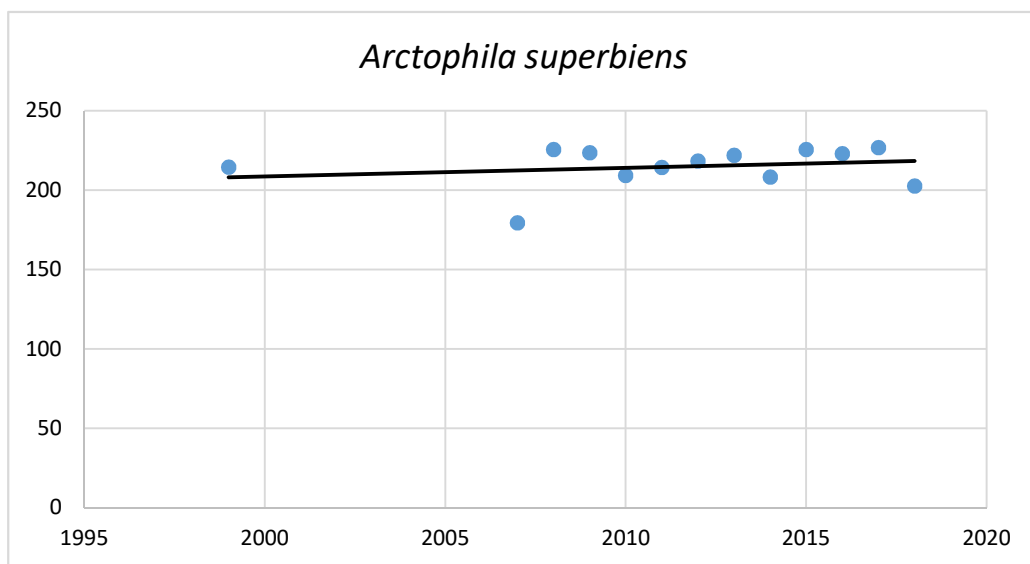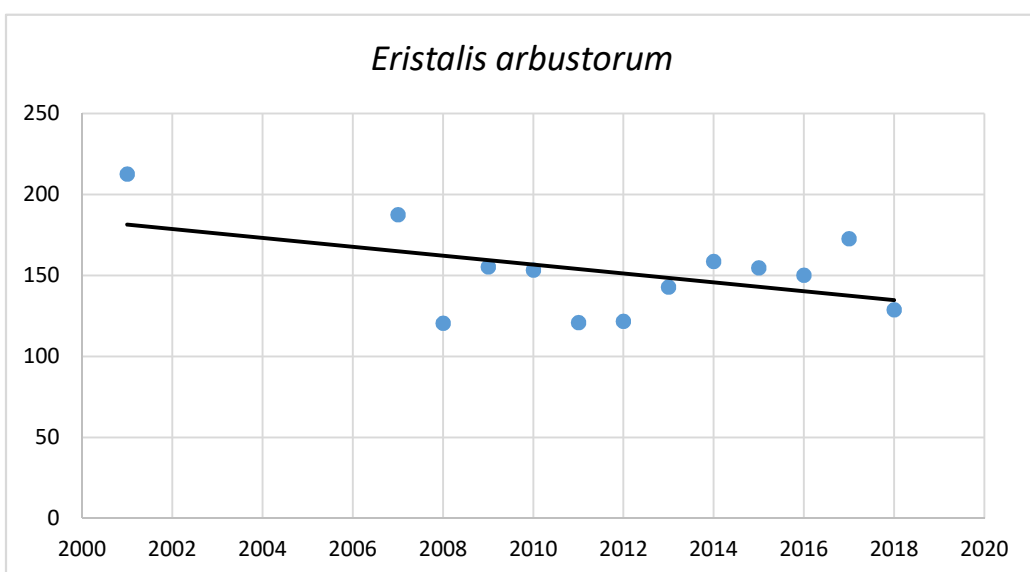

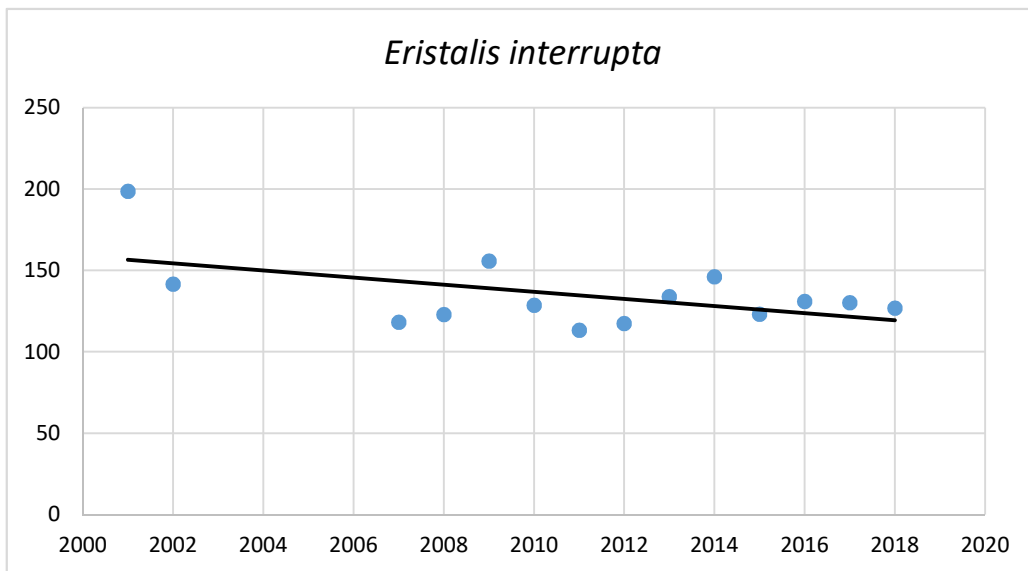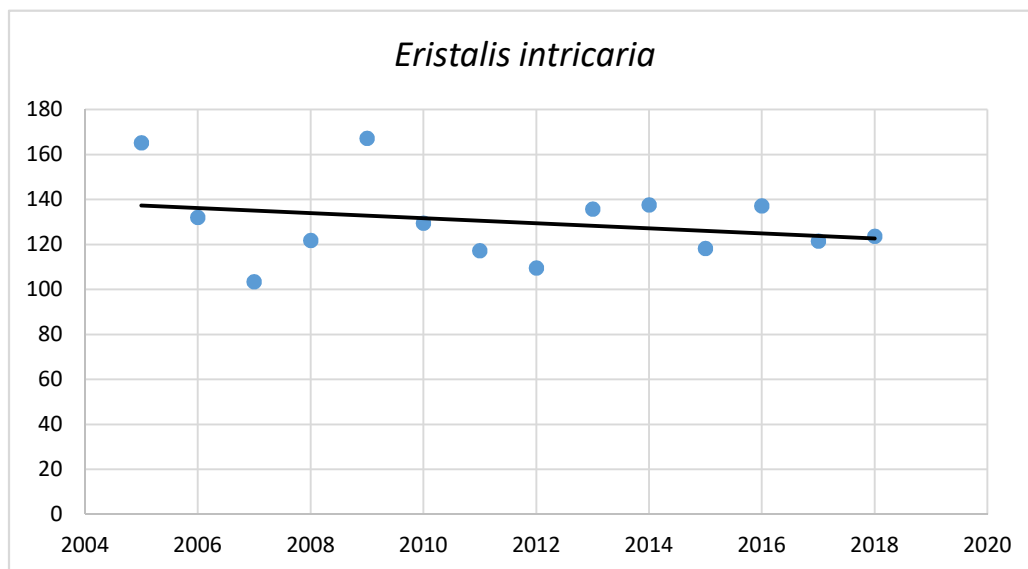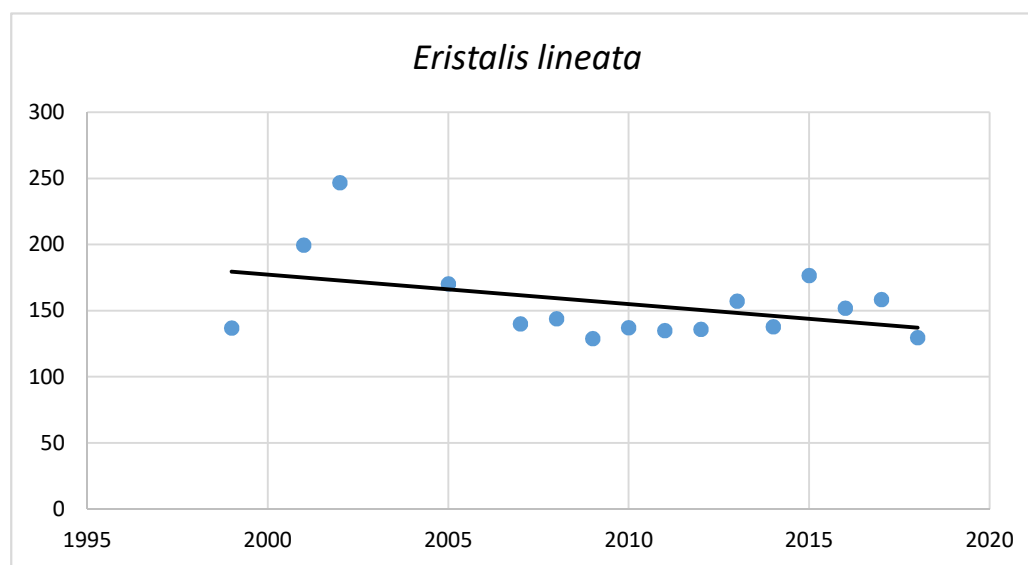

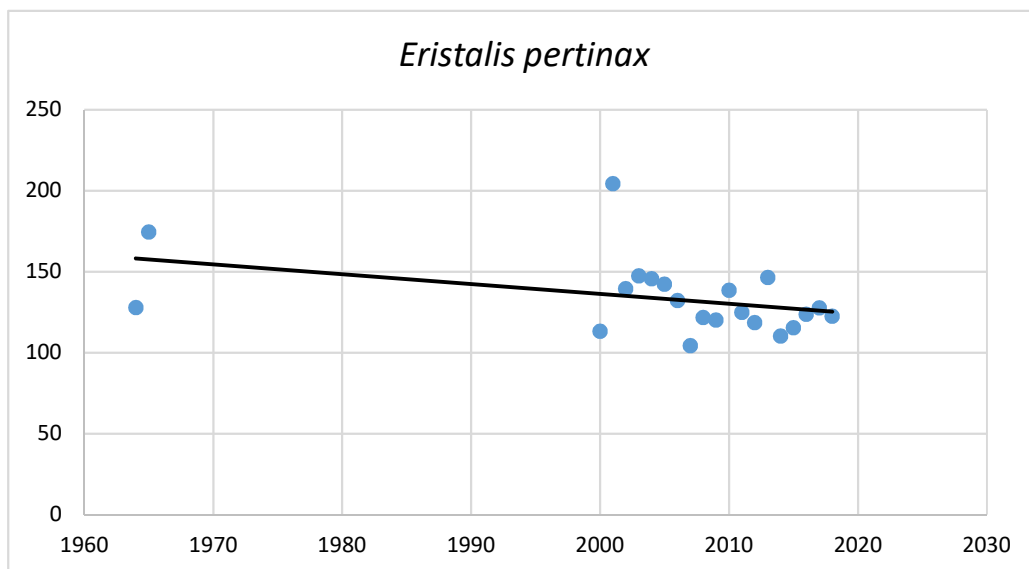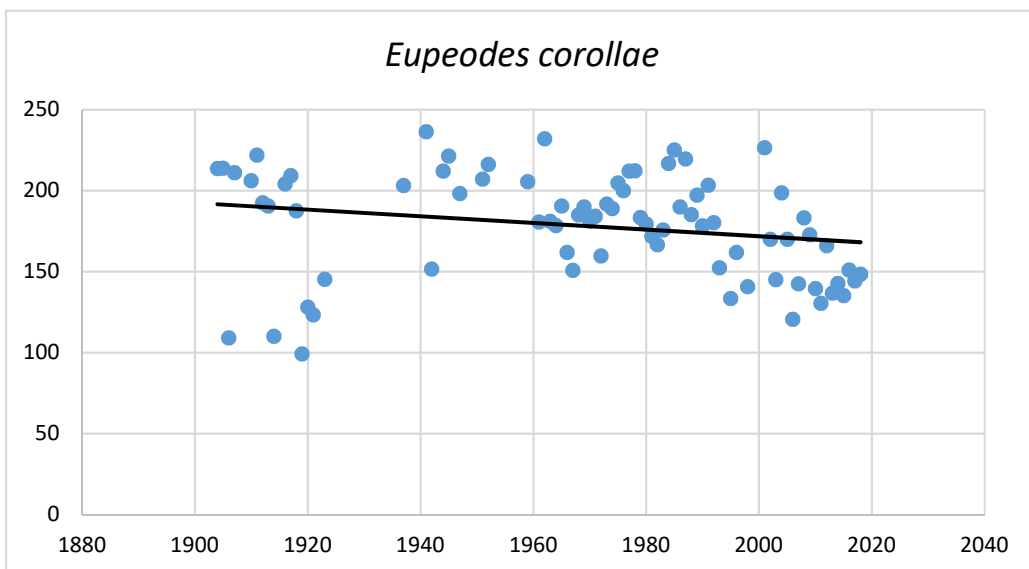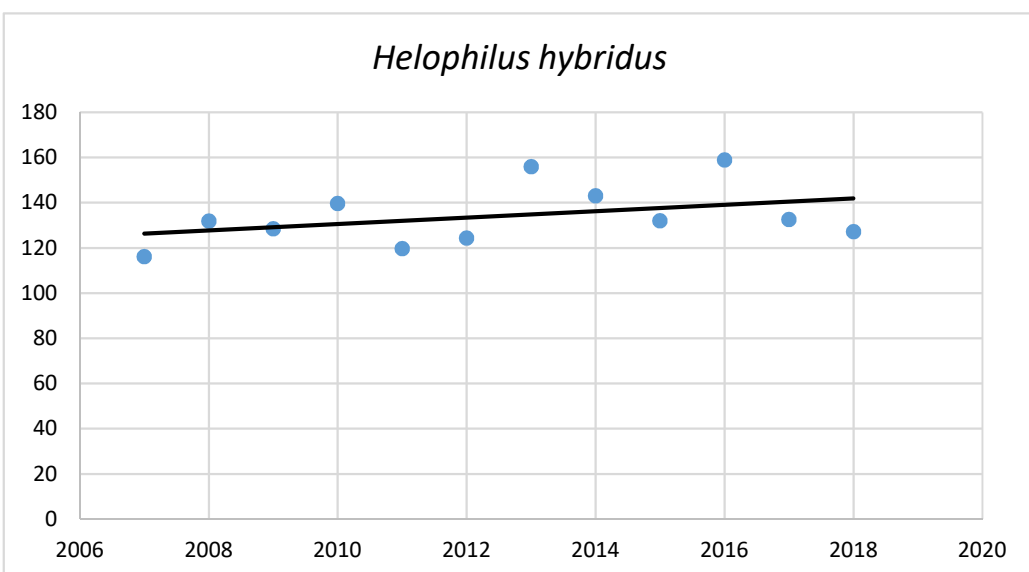

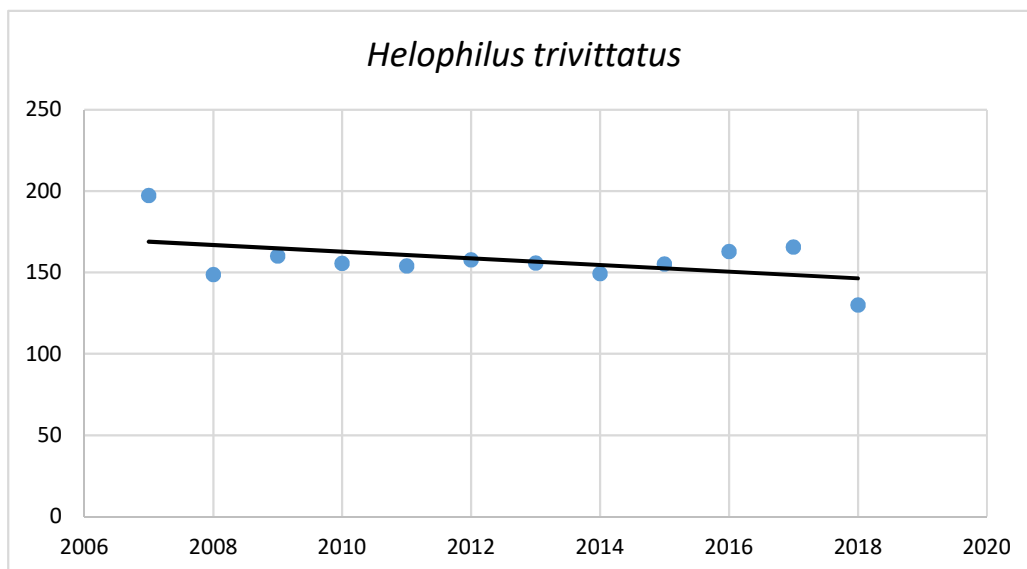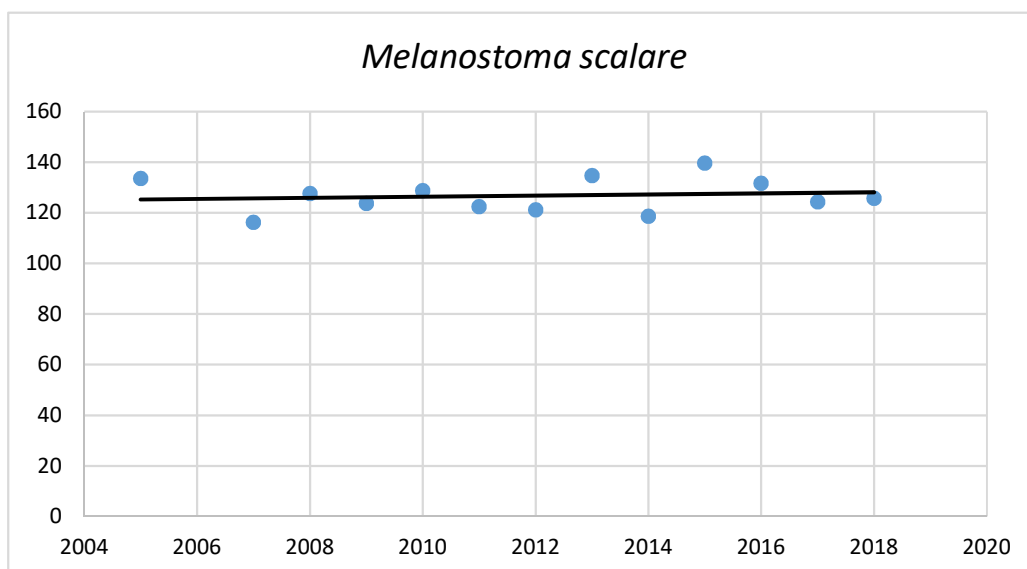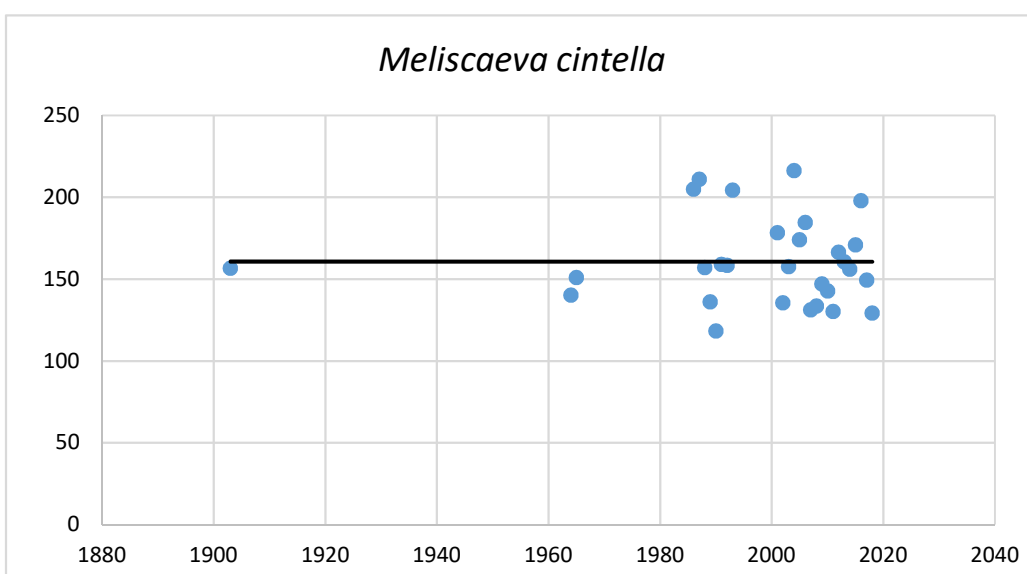

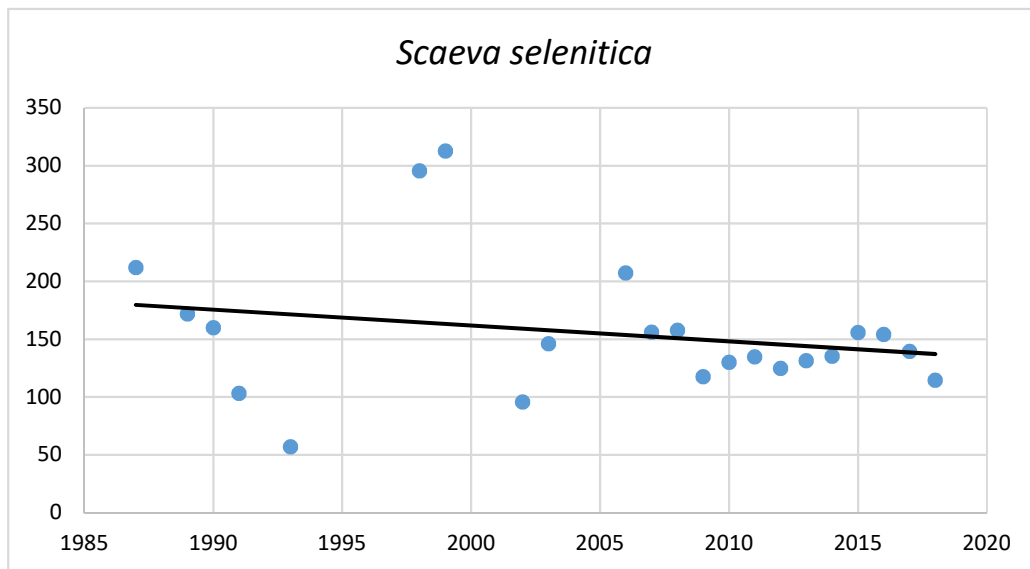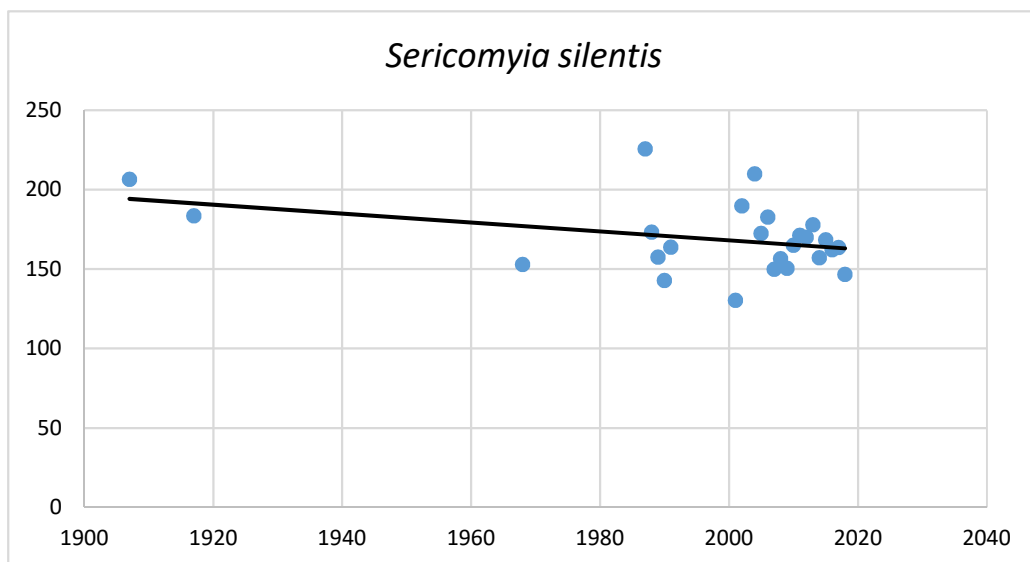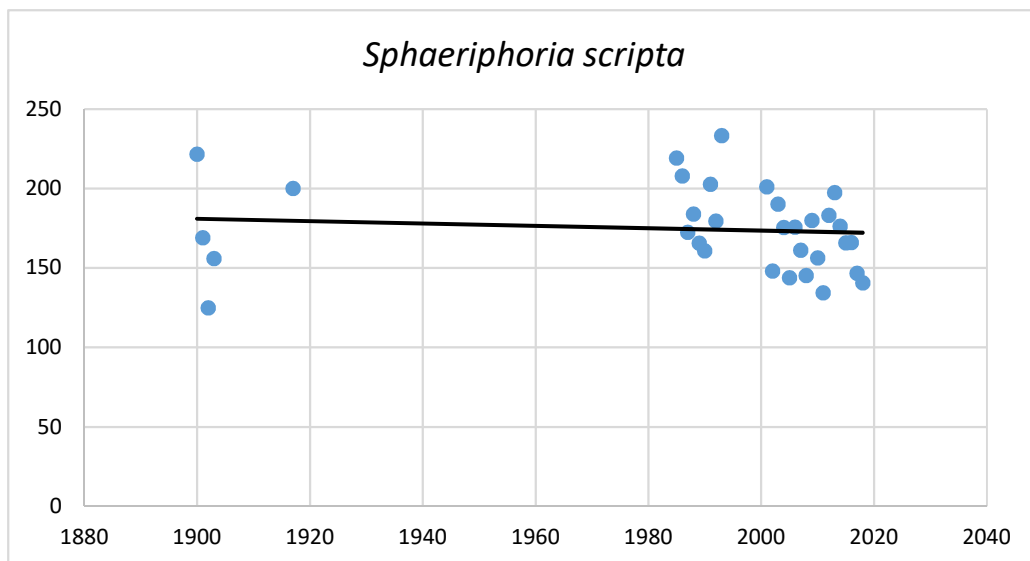

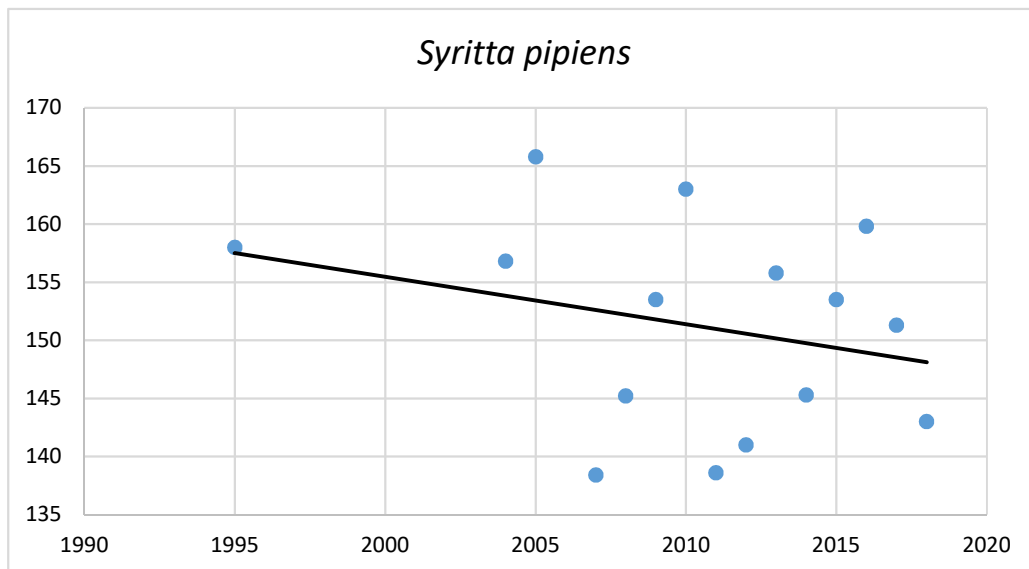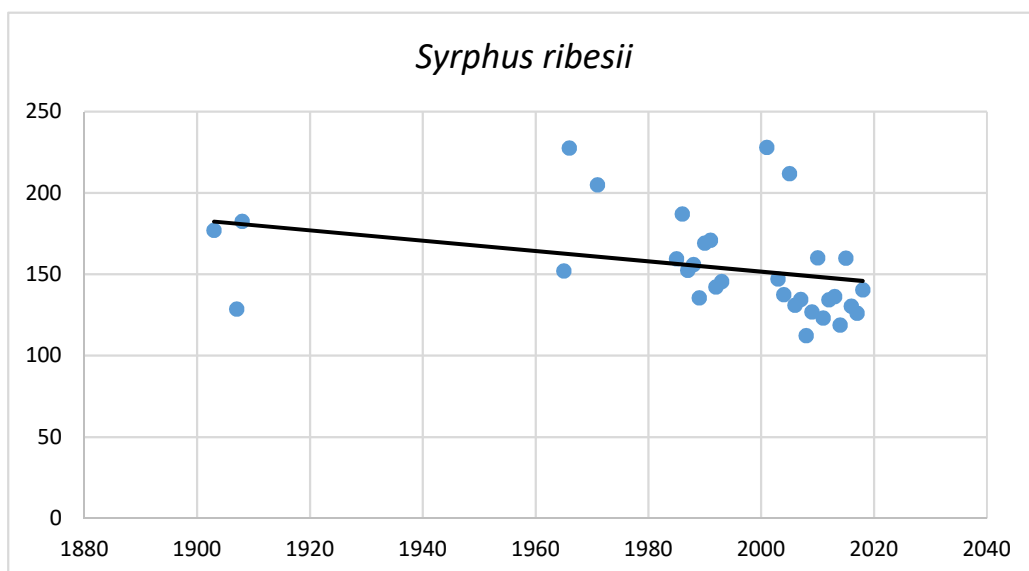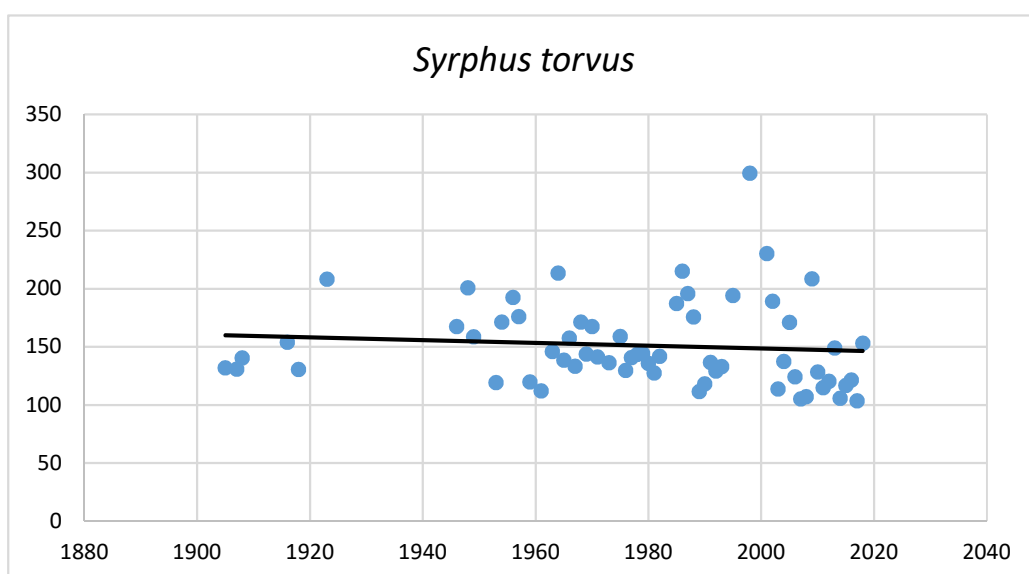

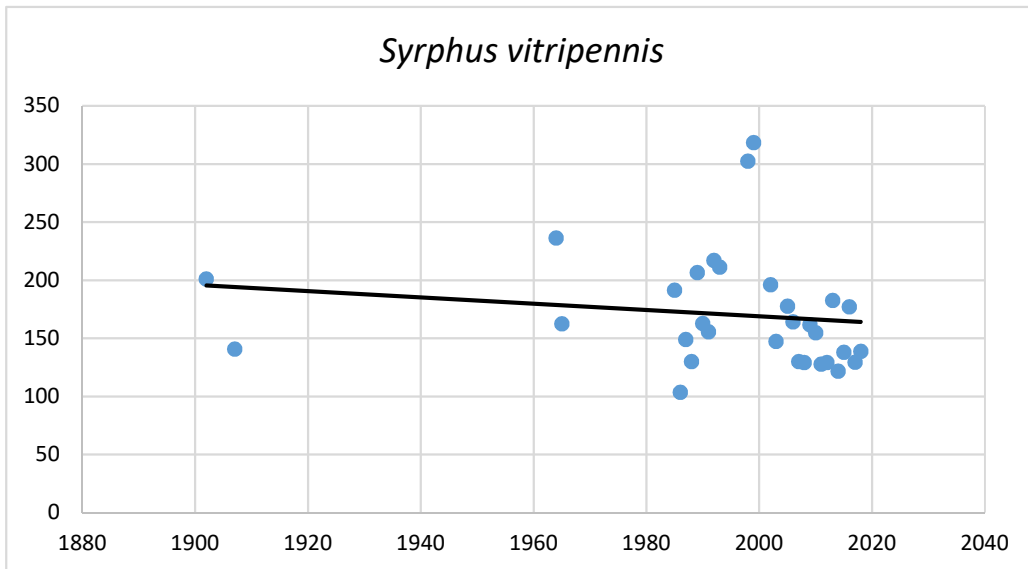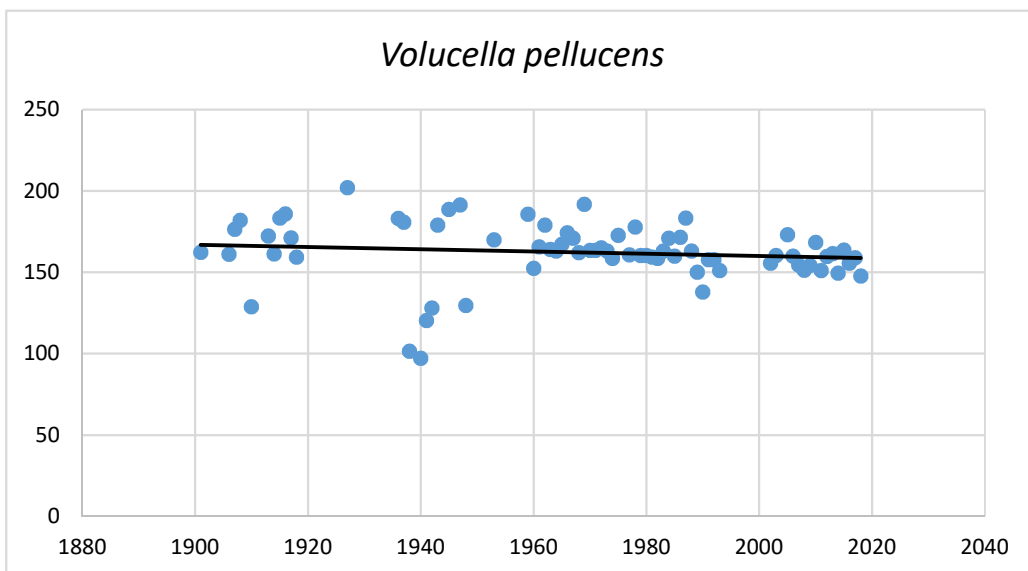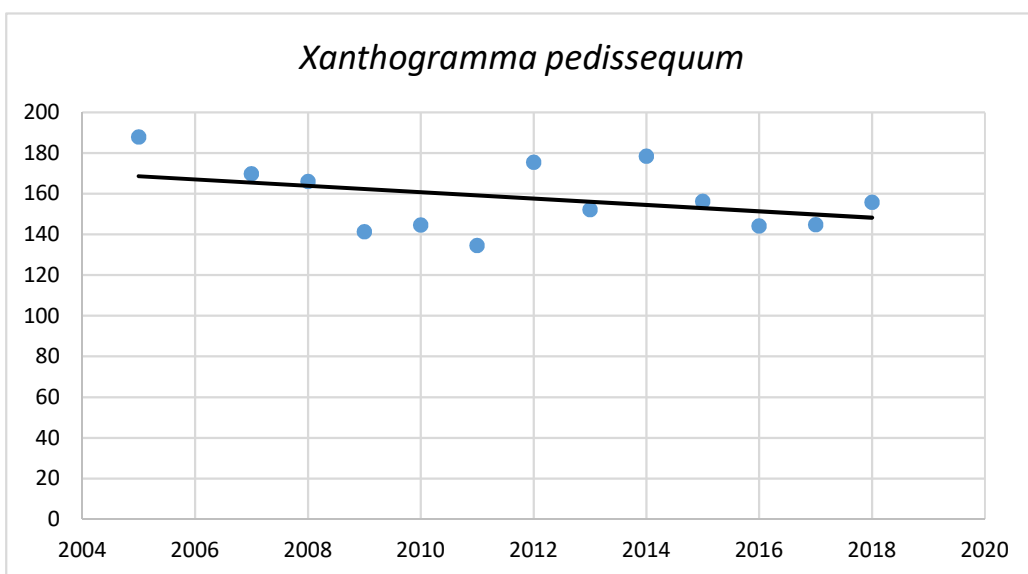

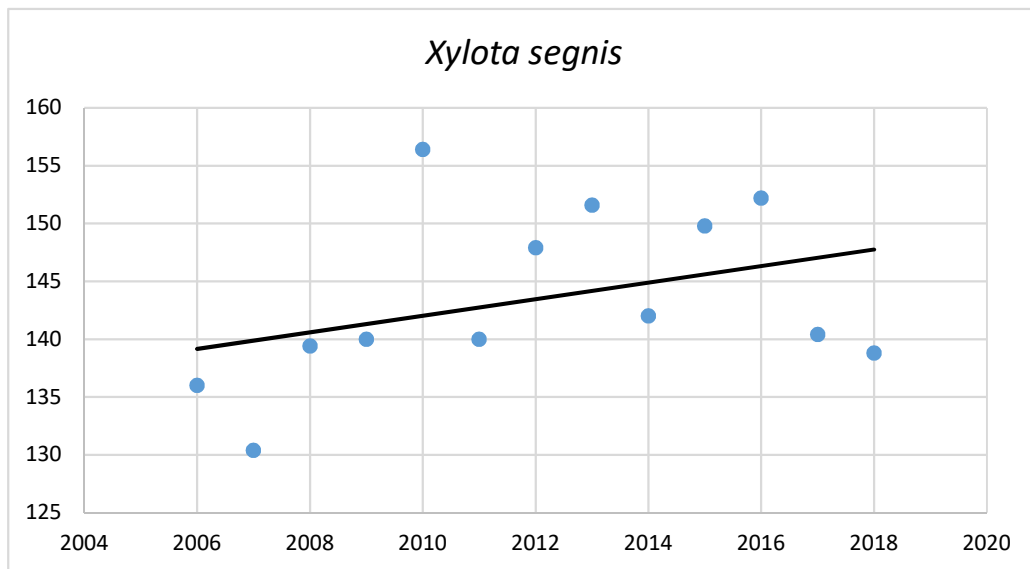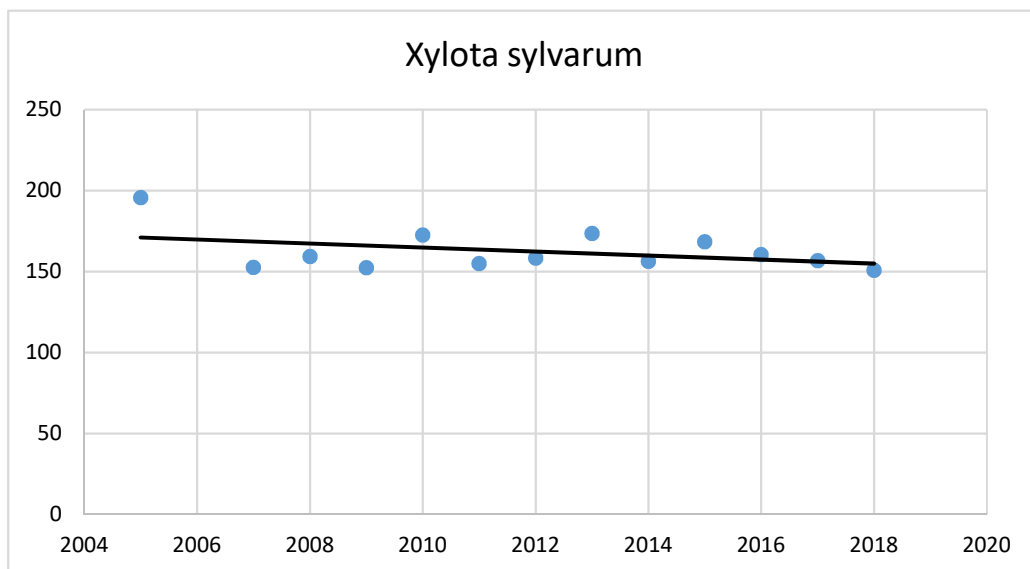

Supplement: S1 Fig — Trend line plots of 10th percentile DOY (y-axis) and year (x-axis) for the 22 species of Danish hoverflies, which did not show a statistically significant correlation between the recorded earliest flight date and the year of observation. p-values, slope coefficient-values and R2-values from the regression analyses can be found in Table 1. (PDF) [file pone.0232980.s002.pdf]
